# 2022 Renal denervation therapy for the treatment of hypertension: a statement from the Thai Hypertension Society

**DOI:** 10.1038/s41440-022-01133-6

**Published:** 2023-02-09

**Authors:** Weranuj Roubsanthisuk, Sirisawat Kunanon, Pairoj Chattranukulchai, Pariya Panchavinnin, Nattawut Wongpraparut, Jarkarpun Chaipromprasit, Pavit Pienvichitr, Rapeephon Kunjara Na Ayudhya, Apichard Sukonthasarn, on behalf of Thai Hypertension Society

**Affiliations:** 1 Division of Hypertension, Department of Medicine, Faculty of Medicine Siriraj Hospital, Mahidol University, Bangkok, Thailand; 2 Division of Cardiovascular Medicine, Department of Medicine, Faculty of Medicine, Chulalongkorn University, King Chulalongkorn Memorial Hospital, Bangkok, Thailand; 3 Division of Cardiology, Department of Medicine, Faculty of Medicine Siriraj Hospital, Mahidol University, Bangkok, Thailand; 4 Division of Cardiology, Department of Medicine, Faculty of Medicine Ramathibodi Hospital, Mahidol University, Bangkok, Thailand; 5 Vichaiyut Hospital, 53 Setsiri Road, Phyathai, Bangkok, Thailand; 6 Cardiology Division, Department of Internal Medicine, Faculty of Medicine, Chiang Mai University, Chiang Mai, Thailand

**Keywords:** Blood pressure, Hypertension, Renal denervation, Statement, Thailand

## Abstract

Hypertension remains a significant risk factor for major cardiovascular events worldwide. Poor adherence to treatment is extremely common in clinical practice, leading to uncontrolled hypertension. However, some patients with resistant hypertension still have uncontrolled blood pressure despite good medical compliance. A specific group of patients also develop adverse reactions to many blood pressure-lowering medications. These scenarios indicate that innovative strategies to lower blood pressure in challenging cases of hypertension are needed. The blood pressure-lowering efficacy of catheter-based renal denervation therapy to decrease sympathetic tone has been confirmed in many publications in recent years. Apart from both the invasiveness and the expensiveness of this technology, appropriate case selection to undergo this procedure is still developing. The utilization of renal denervation therapy for hypertension treatment in Thailand has lasted for 10 years with a good response in most cases. Currently, only certain interventionists at a few medical schools in Thailand can perform this procedure. However, more physicians are now interested in applying this technology to their patients. The Thai Hypertension Society Committee has reviewed updated information to provide principles for the appropriate utilization of renal denervation therapy. The blood pressure-lowering mechanism, efficacy, suitable patient selection, pre- and postprocedural assessment and procedural safety of renal denervation are included in this statement.

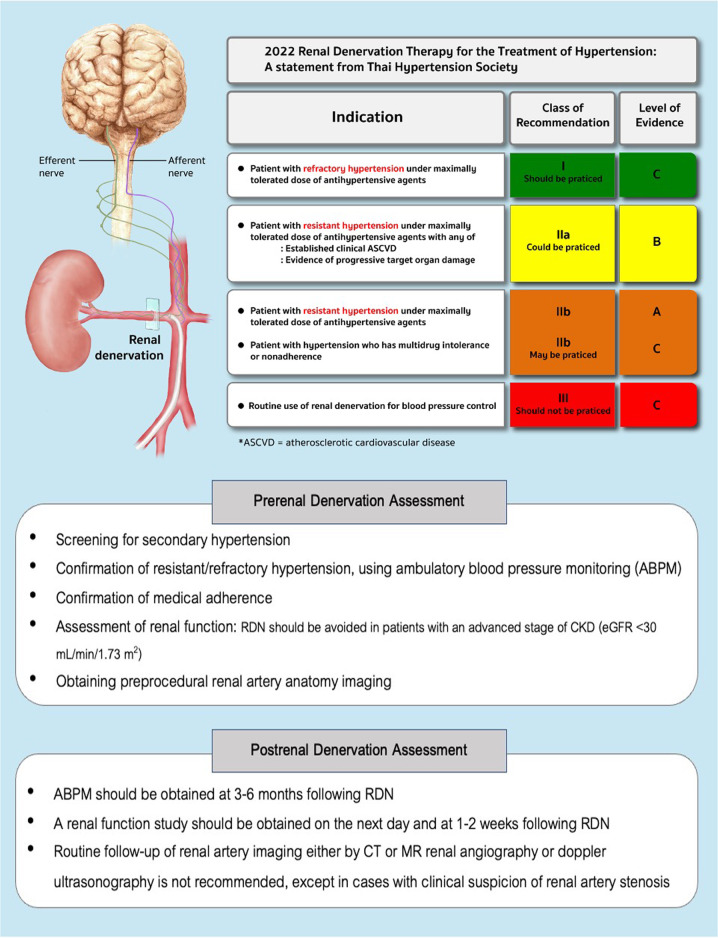

## Introduction

Hypertension is the leading risk factor for major cardiovascular events. The worldwide prevalence of hypertension is 34%, according to the latest survey organized by the International Society of Hypertension among more than 1.5 million people from 92 countries in 2019 [1]. Despite the development of many effective and safe blood pressure (BP)-lowering medications in this era, the rate of achieving BP targets among hypertensive subjects is low in most parts of the world [2, 3]. In particular, poor adherence to treatment is a major cause of treatment failure [4]. However, certain groups of hypertensive patients still have their BP uncontrolled despite their good compliance with many antihypertensive medications. Additionally, some patients may experience significant side effects from certain BP-lowering medications, making BP control even more difficult. Therefore, researchers are still looking for novel strategies or techniques other than medication to lower BP in hypertensive patients. According to the Thai National Health Examination Survey (NHES), the BP control rate among hypertensive subjects receiving treatment in Thailand dropped from 61% in the 5th survey in 2014 to 48% in the 6th survey in 2020 [5, 6]. Data from a nationwide study in Thailand showed that 17% of the hypertensive population was on three or more BP-lowering medications [7]. At the hypertension clinic of one tertiary care university hospital, 33% of patients needed three or more antihypertensive drugs for BP control. This information implies that innovative approaches to help in BP control are also required for the Thai population.

**Fig. 1:**
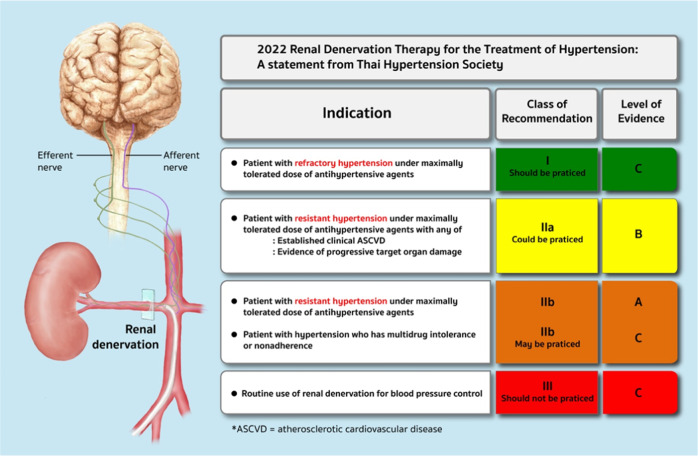
Summary of indications for renal denervation

Catheter-based renal denervation therapy (RDN) was first reported in 2009 by Krum et al. [8]. To date, it has been shown in many studies that RDN can significantly lower BP in humans with hypertension. However, the degree of BP decline after RDN varies among studies. Since RDN is an invasive and costly therapy, appropriate patient selection to undergo this procedure is now being considered. Furthermore, certain groups of physicians and patients are interested in applying RDN for sustained BP control in the long run, with the expectation that long-term BP control could be achieved with the consumption of a lower dosage of antihypertensive medication (Fig. 1).

In Thailand, interventional cardiologists started to apply this technology in 2012 [9]. However, this novel treatment is only performed by interventionists at a few large medical schools. Since there has been much information regarding the progress of RDN technology published recently, the Thai Hypertension Society Committee agreed that a guiding principle for the appropriate utilization of RDN in Thailand is needed. Therefore, the views of experts in hypertension management and RDN interventionists were canvassed to compose a statement about RDN utilization. The strength of the recommendation and the quality of the evidence described in this statement are clarified in Tables 1 and 2.

**Table 1:** Strength of the recommendation

Level I	“Should be practiced”	The recommendation is highly reliable, beneficial to patients, and worthwhile
Level IIa	“Could be practiced”	The recommendation is moderately reliable, likely beneficial to patients, and probably worthwhile
Level IIb	“May be practiced”	The recommendation is not reliable enough, without adequate proof that it is beneficial to patients and is probably not worthwhile, but it will not cause them any harm
Level III	“Should not be practiced” or “Must not be practiced”	The recommendation is not beneficial and will probably cause harm to patients

**Table 2:** Quality of evidence

A	Evidence from various high-quality, randomized controlled trials or evidence from meta-analysis
B	Evidence from at least one high-quality, randomized controlled trial or from a large-scale, non-randomized study with a definitive outcome on the advantages or disadvantages
C	Evidence from other types of high-quality studies; a retrospective descriptive study, a registry study, or agreement among a group of medical specialists based on clinical experience

### How does renal denervation therapy lower blood pressure?

Sympathetic nervous system activation plays a role, in addition to other mechanisms, in BP elevation. In humans, sympathetic afferent and efferent nerve fibers run around the renal arteries within the adventitial layer. The afferent sympathetic nerves transmit signals from the kidneys, usually in response to renal injury, to the hypothalamus, causing an increase in central sympathetic outflow and ultimately BP elevation [10]. The efferent sympathetic nerves exit from the central nervous system and then innervate the kidneys. The main effects of efferent sympathetic nerves on the kidneys are to increase renin secretion, increase sodium reabsorption in the renal tubules, and induce renal vasoconstriction to decrease blood flow to the kidneys [10]. Therefore, attenuating the sympathetic outflow to the kidneys might decrease systemic BP [11]. In the past, before effective BP-lowering medications were widely available, surgical sympathectomy was used to lower BP [12, 13]. This was proof of the concept that a high sympathetic tone is associated with hypertension. However, such a procedure provoked serious postoperative adverse effects [13], and it thus became obsolete with the advent of effective antihypertensive medications. However, the role of renal sympathetic outflow in inducing BP elevation, the anatomy of accessible renal sympathetic nerves, and the need for a novel therapy for hypertension prompted researchers to find other techniques to denervate the sympathetic nerve fiber surrounding the renal arteries. The radiofrequency ablation catheter was first developed to deliver heat to destroy nerves in the adventitial layer of renal arteries. Other techniques to ablate the renal nerve included intravascular ultrasound ablation [14, 15] and alcohol-mediated renal nerve ablation via a Peregrine catheter [16].

### Blood pressure-lowering efficacy of renal denervation therapy in hypertension

**Fig. 2:**
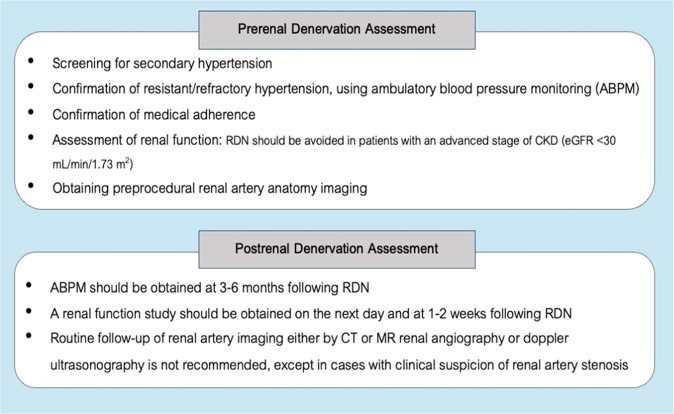
Summary of pre- and post-renal denervation assessment

Initially, data from the first-generation RDN studies showed that RDN effectively reduced BP in patients with resistant hypertension (Table 3). In Thailand, the first report on the efficacy of RDN in patients with resistant hypertension was published in 2014 [9]. The effectiveness of RDN outcomes was maintained for up to 9 years in Thai patients [17]. The strongest predictor of BP reductions following RDN was baseline systolic BP in the post hoc analyses of the SYMPLICITY HTN-3 trial and meta-analysis (Fig. 2).

**Table 3:** First-generation studies of renal denervation

Study	Catheter	Population	Intervention	Comparison	Outcome
SYMPLICITY HTN-1 [77]	SYMPLICITY (radiofrequency device)	Resistant hypertension (≥3 drugs including diuretic, SBP > 160 mmHg)	RDN (*n* = 153)	-	Office BP: - At 1 mo (*n* = 141): −18.9/−9.4 mmHg - At 6 mo (*n* = 144): −22/−10.2 mmHg - At 12 mo (*n* = 132): −26.5/−13.5 mmHg - At 24 mo (*n* = 105): −28.9/−14 mmHg - At 36 mo (*n* = 88): −32/−14 mmHg
SYMPLICITY HTN-2 [78]	SYMPLICITY	Resistant hypertension (≥3 drugs including diuretic, SBP > 160 mmHg)	RDN (*n* = 52)	Same med (*n* = 54)	Office SBP at 6 mo: −32/−12 vs. +1/0 mmHg (*p* < 0.0001)
SYMPLICITY HTN-3 [24, 26]	SYMPLICITY	Resistant hypertension (≥3 drugs including diuretic, SBP > 160 mmHg)	RDN (*n* = 364)	Sham (*n* = 171)	At 6 mo: [26] Office BP : −14.13 ± 23.93 vs −11.74 ± 25.94 mmHg (*p* = 0.26) 24-h SBP: −6.75 ± 15.11 vs. −4.79 ± 17.25 mmHg (*p* = 0.98) At 3 yr: [24] Office BP: −26.4 ± 25.9 vs −5.7 ± 24.4 mmHg (*p* < 0.0001) 24-h SBP: −15.6 ± 20.8 vs. −0.3 ± 15.1 mmHg (*p* < 0.0001)
DENERHTN [79]	SYMPLICITY	Indapamide 1.5 mg, ramipril 10 mg (or irbesartan 300 mg), and amlodipine 10 mg daily for 4 weeks → uncontrolled ABPM	RDN	Sequential adding: spironolactone 25 mg/d, bisoprolol 10 mg/d, prazosin 5 mg/d, rilmenidine 1 mg/d	Daytime SBP at 6 mo: −15.8 vs. −9.9 mmHg (*p* = 0.0329)
Global SYMPLICITY Registry [22]	SYMPLICITY	Severe treatment-resistant hypertension was defined as office SBP ≥ 160 mmHg and 24-h SBP ≥ 135 mmHg, despite the prescription of ≥3 antihypertensive drugs, while “less severe hypertension” was defined as office BP 150–180/≥90 mmHg and 24-h SBP 140–170 mmHg	RDN (*n* = 1742)		Office SBP at 6 mo: −12.8 ± 26.2 mmHg 24-h SBP at 6 mo: −7.2 ± 17.8 mmHg (*p* < 0.0001) The reduction in both office and 24-h BP was sustained at 12, 24, and 36 mo

*BP* blood pressure, *SBP* systolic blood pressure, *RDN* renal denervation therapy, *ABPM* ambulatory blood pressure monitoring

It was reported that RDN could effectively reduce BP over 24 h, including over the nighttime period, which had a strong correlation with organ damage and cardiovascular diseases [18]. The nighttime BP-lowering efficacy of RDN may be beneficial to Thai patients because one report showed that the nighttime BP in Thai patients was higher than that in a Japanese population, despite there being no difference in office BP between these two groups of patients [19].

In patients with resistant hypertension, not only does RDN lead to BP reduction, but it can also improve target organ damage, e.g., reduction of the left ventricular mass and improvement of the left ventricular ejection fraction and diastolic function [20, 21].

After SYMPLICITY HTN3, many RDN devices were developed, and subsequent studies (second-generation studies) usually included a sham-controlled group and assessed BP change by ambulatory BP monitoring (ABPM). The second-generation studies showed that RDN also effectively lowered the BP in patients with less severe than resistant hypertension or patients not taking antihypertensive drugs, as summarized in Table 4.

**Table 4:** Second-generation studies of renal denervation

Study	Catheter	Population	Intervention	Comparison	Outcome
SPYRAL HTN-OFF MED [80]	SPYRAL (multielectrode radiofrequency device)	Office SBP: 150–180 mmHg Office DBP: ≥90 mmHg 24-h SBP: 140–170 mmHg; No antihypertensive drugs	RDN (*n* = 38)	Sham (*n* = 42)	At 3 mo: 24-h SBP: −5.5 vs. −0.5 mmHg 24-h DBP: −5.3 vs. −0.4 mmHg Office SBP: −10 vs. −2.3 mmHg Office DBP: −5.3 vs. −0.3 mmHg
SPYRAL HTN-ON MED [23, 52]		Office SBP: 150–180 mmHg Office DBP: > 90 mmHg 24-h SBP: 140–170 mmHg; On 1–3 antihypertensive drugs	RDN (*n* = 38)	Sham (*n* = 42)	At 6 mo: [52] 24-h SBP: −7·4 (−12.5 to −2.3) mmHg, *p* = 0.0051 24-h DBP: −4·1 (−7.8 to −0.4) mmHg, *p* = 0.0292 Office SBP: −6·8 (−12.5 to −1.1) mmHg, *p* = 0.0205 Office DBP: −3·5 (−7.0 to 0) mmHg, *p* = 0.0478 At 3 yr: [23] 24-h SBP: −10 (−16.6 to −3.3) mmHg, *p* = 0.0039 Morning SBP: −11 (−19.8 to −2.1) mmHg, *p* = 0.016 Daytime SBP: −8.9 (−16.5 to −1.2) mmHg, *p* = 0.024 Nighttime SBP: −11.8 (−19 to −4.7) mmHg, *p* = 0.0017 Office SBP: −8.2 (−17.1 to 0.8) mmHg, *p* = 0.073
SPYRAL HTN-OFF MED Pivotal [81]	SPYRAL	Office SBP: 150–180 mmHg	RDN (*n* = 166)	Sham (*n* = 165)	At 3 mo: 24-h SBP: −3.9 (−6.2 to −1.6) mmHg Office SBP: −6·5 (−9·6 to −3·5) mmHg
RADIANCE-HTN SOLO [14]	ReCor Medical Paradise System (ultrasound device)	Daytime BP ≥ 135–170/85–105 mmHg after 4-week discontinuation of up to 2 antihypertensive drugs	RDN (*n* = 74)	Sham (*n* = 72)	At 2 mo: Daytime SBP: −8.5 vs. −2.2 mmHg (*p* = 0.0001)
RADIANCE-HTN TRIO [15]	ReCor Medical Paradise System	Office BP ≥ 140/90 mmHg despite ≥3 antihypertensive drugs, including a diuretic → Eligible patients were switched to a once-daily, fixed-dose SPC of a CCB, an ARB, and a thiazide diuretic After 4 weeks of standardized therapy, patients with daytime BP of ≥135/85 mmHg	RDN (*n* = 69)	Sham (*n* = 67)	At 2 mo: Daytime SBP: −8.0 vs. −3.0 mmHg
REQUIRE [82]	ReCor Medical Paradise System	Seated office BP ≥ 150/90 mmHg despite treatment with a stable regimen, including the maximum tolerated dose of at least three antihypertensive drugs from different classes (including a diuretic) and 24-h SBP of ≥140 mmHg	RDN (*n* = 72)	Sham (*n* = 71)	At 3 mo: 24-h SBP: −6.6 vs. −6.5 mmHg (*p* = 0.971)

*SBP* systolic blood pressure, *DBP* diastolic blood pressure, *RDN* renal denervation therapy, *SPC* single-pill combination, *CCB* calcium channel blocker, *ARB* angiotensin II receptor blocker

The BP reduction effect of RDN in humans is durable for at least 3 years, as shown in the Global SYMPLICITY registry [22], SPYRAL HTN-ON MED study [23], SYMPLICITY HTN-3 trial [24] and a report from Thailand [17]. In the SYMPLICITY HTN-3 trial [24], RDN induced a significant reduction in both office systolic BP (−26.4 versus −5.7 mmHg, *p* < 0.0001) and 24-h ambulatory systolic BP (−15.6 versus −0.3 mmHg, *p* = 0.0001) compared to the sham control at the 3-year follow-up. Long-term follow-up in 18 Thai subjects receiving RDN has shown at least 80% effectiveness of RDN, defined by a reduction in office systolic BP > 10 mmHg, a reduction in the number of antihypertensive drugs taken, or both. The mean and longest follow-up periods in our study were 52 months and 104 months, respectively [17]. Therefore, we still need future studies concerning renal nerve regeneration, BP lowering, and safety information related to RDN in the future.

### Which hypertensive patients should be treated with RDN?

For the current situation in Thailand, the committee responsible for this statement endorsed that RDN should be considered in hypertensive patients with the following conditions:

1. refractory hypertension under the maximally tolerated dose of antihypertensive drugs *(Strength of Recommendation I, Quality of Evidence C)*
2. resistant hypertension under the maximally tolerated dose of antihypertensive drugs with:
  - established clinical atherosclerotic cardiovascular disease (ASCVD) *(Strength of Recommendation IIa, Quality of Evidence B)*
  - evidence of progressive target organ damage *(Strength of Recommendation IIa, Quality of Evidence B)*
3. resistant hypertension under the maximally tolerated dose of antihypertensive drugs *(Strength of Recommendation IIb, Quality of Evidence A)*
4. multidrug intolerance or nonadherence *(Strength of Recommendation IIb, Quality of Evidence C)*.

Refractory hypertension is generally defined as uncontrolled hypertension despite the use of ≥5 different classes of antihypertensive drugs, including a long-acting thiazide or thiazide-like diuretic (e.g., chlorthalidone) and a mineralocorticoid receptor antagonist (e.g., spironolactone or eplerenone) [25]. Resistant hypertension means hypertension that has remained uncontrolled despite using ≥3 antihypertensive drugs, including if tolerated, a diuretic [25].

Patients with resistant hypertension were the first group in whom the role of RDN was assessed. Among those with resistant hypertension, there were many patients whose BP was still high despite the use of ≥5 different classes of antihypertensive drugs. For example, in the SYMPLICITY HTN-3 trial [26], the number of antihypertensive medications at baseline was 5.1 ± 1.4 and 5.2 ± 1.4 in the RDN and sham-operated groups, respectively. On average, four BP-lowering medications were at maximally tolerated dosages [26]. This refractory subtype of resistant hypertension is less likely to be caused by poor compliance or adherence, which are common in general cases of resistant hypertension. If more medications are added, such patients will be exposed to various adverse drug effects.

Given the very high-risk nature of this specific subtype of hypertension, these patients should have an RDN procedure performed (I, C).

Data from randomized controlled trials and registries have proven that RDN is safe and effective for patients with resistant hypertension, and the effects are sustained for at least 36 months [24]. This effectiveness is consistent across different populations, including high-risk subgroups, and independent of the number of prescribed antihypertensive medications. Considering the invasive nature of the RDN procedure and the possibility of poor medical compliance in a significant number of patients with resistant hypertension [27], we considered the priority of performing RDN only for those receiving a maximally tolerated dose of antihypertensive drugs. Patients presenting resistant hypertension with established clinical ASCVD and with evidence of progressive target organ damage (TOD) have the highest risk of future cardiovascular complications, and they could be considered for RDN (IIa, B). Patients with resistant hypertension confirmed to have been treated using a maximally tolerated dose of antihypertensive drugs but without clinical ASCVD and without progressive TOD may be considered for RDN (IIb, A). Although the effectiveness and safety of RDN are considered to be similar and independent of individual ASCVD risk, patients without clinical ASCVD and without progressive TOD are considered in less urgent clinical conditions; thus, the strength of recommendation is IIb. A cost-effectiveness study of RDN in resistant hypertension from Australia indicates that RDN would be cost-effective only if it was initially targeted to patients whose 10-year predicted cardiovascular risk was at least 13.2% [28].

Patients without resistant hypertension but with multidrug intolerance or nonadherence are difficult to treat pharmacologically. Given the invasive nature of the RDN procedure, the lack of information on the reduction of cardiovascular events and the lack of a cost-effectiveness analysis in Thai patients, these patients may also be considered for RDN (IIb, C).

RDN trials and registries have suggested that RDN should not be reserved only for patients with resistant HT; however, no clinical trial strongly recommends using RDN as a standard first-line treatment for HT. Therefore, RDN should be considered a complementary BP-lowering strategy in situations where BP targets are difficult to achieve and maintain and should not be used routinely in every hypertensive patient (III, C).

The routine use of RDN for BP control in hypertensive patients is still not recommended *(Strength of Recommendation III, Quality of Evidence C)* since RDN is associated with certain complications, and evidence regarding its efficacy for long-term BP control is needed. In addition, RDN is not recommended for BP lowering in patients with secondary hypertension or an estimated glomerular infiltration rate (eGFR) <30 mL/min/1.73 m^2^.

### Which kinds of patients are more likely to benefit from RDN?

Previous clinical studies have shown that specific clinical parameters, including pre-RDN BP level [29], diastolic BP variability [30], combined systolic–diastolic hypertension [31], 24-h ambulatory heart rate (>74 beats/min) [32], renal artery vasodilatation [33], aortic pulse wave velocity [34], central pulse pressure [35], younger vascular age [36], low abdominal aortic calcification burden [37], and impaired cardiac baroreflex sensitivity [38], were potential predictors of RDN responders. However, this information has significant limitations due to the retrospective nature of the analysis and significant differences in baseline demographic variables, including BP, age, and comorbidities. Thus, the hypothesis that RDN is more or less effective in certain groups of patients remains unproven and warrants further investigation.

### Prerenal denervation assessment

#### Screening for secondary hypertension

- *RDN is not recommended for BP control in patients with known secondary hypertension*.

Identifiable causes of hypertension can be found in ~10% of patients (with a greater percentage of patients with resistant hypertension). The common causes of secondary hypertension are renal parenchymal disease, renovascular disease, primary aldosteronism, and substance/drug-induced hypertension. Not all hypertensive patients should be evaluated for secondary hypertension. Essential clues for suggesting a secondary condition are (a) young patient <40 years of age, (b) moderate to severe hypertension (systolic BP ≥ 160 or diastolic BP ≥ 100 mmHg) or resistant hypertension, and (c) symptoms or signs suggesting secondary causes [39]. Patients with known secondary hypertension, such as those with primary aldosteronism, Cushing’s syndrome, or renal artery stenosis, have generally been excluded from RDN trials and registries; thus, data on the efficacy of RDN in these groups of patients are lacking. Nonetheless, some evidence demonstrates that RDN is effective and safe in patients with obstructive sleep apnea or moderate chronic kidney disease (CKD) [40–44]. In patients with renal artery stenosis whose BP remains uncontrolled despite renal artery revascularization, RDN in a plaque-/stent-free segment could be performed without complications. However, there is little information available from specific case reports [45–47]. Therefore, the committee does not recommend RDN for BP lowering in this group of patients.

#### Confirmation of uncontrolled hypertension/resistant hypertension using ambulatory blood pressure monitoring

- *BP measurement using ABPM is recommended for all candidates prior to the RDN procedure*.

White coat hypertension occurs when patients have high office BP but normal out-of-office BP. Some patients may be misdiagnosed if only office BP is used for clinical assessment. In the recently published second generation of the RDN trials, ABPM was routinely obtained to ensure the status of BP control. Home BP monitoring (HBPM) plays a role in the diagnosis and monitoring of hypertension treatment. It helps to remind patients to take their antihypertensive medications regularly, thus leading to better BP control. However, HBPM requires the patient’s capability to obtain reliable BP records compared with ABPM [48]. According to recent RDN trials, the committee recommends obtaining ABPM in all candidates prior to the RDN procedure.

### Confirmation of medical adherence

One of the causes of uncontrolled hypertension is nonadherence to medical treatment. Using biochemical screening, nonadherence to antihypertensive drugs has been identified in ~30–50% of patients receiving treatment for hypertension [49–51]. According to data from SPYRAL HTN-ON MED, up to 42.5% of participants were classified as nonadherent during the intensive follow-up period [52]. The prevalence of nonadherence to medications is also high among Thai hypertensive populations, ranging from 40 to 87% in recently published studies [53–55]. Since improved medical adherence will lead to better BP control and a reduced risk of cardiovascular events and all-cause mortality [56], physicians should encourage their patients to maximize their adherence to antihypertensive medication before considering device-based therapy.

Single-pill combination drugs and polypills have been recommended to improve medication adherence and BP control in hypertension [27]. Thai Hypertension Society guidelines on the treatment of hypertension also suggest using single-pill combination drugs for treating hypertension in the Thai population [39]. However, the prescription of single-pill combination drugs is extremely low in governmental hospitals of different sizes in Thailand [57]. Therefore, using single-pill combination drugs is also encouraged to improve medication adherence.

### Assessment of renal function: serum creatinine and estimated glomerular infiltration rate

- *Renal function studies should be performed during treatment planning*.
- *RDN should be avoided in patients with an advanced stage of CKD (eGFR* < *30* *mL/min/1.73* *m*^2^*)*.

CKD is common among patients with hypertension, either as a cause or sequelae. Data from a nationwide survey in Thailand indicated that 29% of individuals with uncontrolled hypertension had eGFR <60 mL/min/1.73 m^2^ [6]. Blood testing for serum creatinine and eGFR can be used for the screening of CKD and for classifying its severity. Most of the randomized control trials in RDN have excluded patients with eGFR <45 mL/min/1.73 m^2^. Although a few small single-center, nonrandomized studies have reported on the safety and effectiveness of RDN in patients with CKD stage 3–4, the current data are insufficient to recommend this procedure in patients with an advanced stage of CKD [43, 44]. The committee agrees that CKD patients with eGFR ≥30 mL/min/1.73 m^2^ could be considered RDN candidates if all the standard methods for preventing contrast-induced nephropathy are employed.

### Renal artery anatomy imaging [computed tomography (CT) or magnetic resonance (MR) renal angiography]

- *Preprocedural renal artery anatomy imaging by either CT or MR renal angiography should be obtained to identify the ostia location, accessory renal arteries, abnormal anatomy, or any stenotic lesion of the renal arteries*.

The main renal arteries generally originate from the abdominal aorta at the level between the upper margin of the L_1_ to the lower margin of the L_2_ vertebrae. The right main renal artery origin is usually superior to the left main renal artery origin. In one study, ~70–80% of individuals had a bilateral single renal artery [58]. Renal artery variations, divided into early division and extrarenal arteries, have been observed in 20–30% of the general population [59, 60]. The prevalence of accessory renal arteries was 12% in patients who underwent RDN at Siriraj Hospital [17]. RDN can be performed in a renal artery with a diameter of 3–8 mm. Unawareness of the accessory renal artery can lead to incomplete sympathetic nerve ablation. In patients with accessory renal arteries, BP reduction was more pronounced in a completely denervated accessory artery group than in an incompletely denervated accessory artery group [61].

Preprocedural imaging using CT or MR angiography to identify the location of the renal artery ostia, the presence of accessory branches, or any stenotic lesion will help to screen for a suitable renal artery anatomy and may decrease the procedural time. In the presence of renal artery stenosis, RDN should not be performed. Revascularization with angioplasty and stenting should be considered as a treatment option in RAS associated with poorly controlled hypertension or deterioration of renal function. If there is no preprocedural imaging, an abdominal aortogram should be performed to identify the accessory renal artery and whether there is an unfavorable anatomy before selective engagement of the renal artery for RDN.

### Renal denervation procedural safety

- *RDN should be avoided in patients with an unsuitable anatomy of the renal artery/access site or any condition that would increase the risk of the procedure, such as a bleeding disorder*.

The RDN procedure is generally performed under local anesthesia and conscious sedation to lessen pain. In most clinical trials, the renal artery can be successfully accessed via a femoral approach. Periprocedural adverse and unexpected events within 30 days of the procedure are rare. In our series of 18 patients who underwent RDN at Siriraj Hospital, one patient had renal artery spasms after the procedure, which was successfully treated with intraarterial nitroglycerine. There were no long-term complications after RDN, with the longest follow-up extending up to 9 years [17].

RDN should be avoided in patients with an unfavorable renal artery anatomy. RDN in patients with a heavily calcified, tortuous abdominal aorta, aortic aneurysm, or prior aortic dissection would be difficult and dramatically increase the risk of serious complications. Other contraindications for RDN are similar to those in coronary angiography, such as an increased bleeding risk (bleeding diathesis, thrombocytopenia), advanced stage of CKD, pregnancy, and previous renal intervention (angioplasty, stent implantation) [62].

### Procedural optimization

- *Methods capable of providing completeness of denervation should be ensured for the maximal effect of RDN, including*

- *circumferential ablation*
- *an adequate number of total ablations*
- *distal branch and accessory renal artery ablations*

In SYMPLICITY HTN-3, where researchers were unable to prove the superiority of RDN over the sham control, many limitations, which may be obstacles to effective BP lowering by the procedure, were widely discussed [26]. Kandzari et al. suggested that 4-quadrant ablations in both renal arteries resulted in greater BP reduction than 4-quadrant ablations on one side or in the sham control [63]. Animal model studies have shown that complete 4-quadrant ablations provide circumferential ablation of the renal sympathetic nerve surrounding the renal artery, resulting in a reduction in renal tissue norepinephrine concentrations [64, 65].

The total number of ablations also influences the outcome of RDN. In one study, the total number of ablations predicted office systolic BP reduction at 6 months [63]. In that study, in patients with fewer than nine total ablations, office systolic BP increased 12 mmHg compared with the sham operation. In patients who received more than 14 total ablations, the office systolic BP decreased by 14 mmHg compared with the sham procedure. The trend was significant in the correlation between the total number of ablations and systolic BP reduction. However, the report did not recommend a specific total number of ablations. Inadequate ablation would lead to incomplete denervation and suboptimal BP reduction. On the other hand, too many ablations could theoretically lead to hypotension and increased complications. RDN devices, such as “Simplicity Spyral”, emit radiofrequency to generate heat for nerve ablation. Repeated ablation at the same point could theoretically lead to perforation or stricture. Therefore, the operator should have experience or adequate training for this specific task.

Human autopsy studies have revealed that the distance between the sympathetic nerve surrounding the renal arteries and vessel wall decreases from the proximal to the distal segment [66, 67]. Additionally, the renal sympathetic nerve is closer to the lumen in the distal part of the vessel. Renal sympathetic nerve ablation will be more effective when ablation is performed in the distal portion of the vessel than when it is performed in the proximal region. Mahfoud et al. found a more significant reduction in the renal norepinephrine concentration when combined ablation was performed in both main and branch vessels compared with ablation performed only in main or branch vessels alone in a porcine model [68]. Similar findings were also observed in a human study, with greater BP reduction achieved in subjects with both main and branch vessel ablation [69].

The presence of accessory renal arteries also affects the degree of BP reduction. In patients with accessory renal arteries, BP reduction was found to be more pronounced in a completely denervated accessory artery group than in an incompletely denervated accessory artery group [61]. However, the improvement in 24-h systolic BP was significantly less in subjects with accessory renal arteries than in those without accessory renal arteries [61]. This result emphasizes the importance of complete denervation of all procedure-capable renal arteries.

Many technical aspects mentioned above help optimize the RDN procedure. However, the effect of RDN on BP reduction cannot be confirmed during the procedure with the current technology. Currently, the interventionist will not receive feedback from the RDN device system regarding the technical success of the procedure [11]. Therefore, the degree of denervation may vary among cases, leading to variable BP responses. A means for validation of adequate renal sympathetic nerve ablation during the operation remains to be developed.

### Postrenal denervation assessment

- *ABPM values should be obtained at 3–6 months following RDN*.
- *A renal function study, including serum creatinine and eGFR, should be performed on the next day and at 1–2 weeks following RDN*.
- *Routine follow-up of renal artery imaging either by CT or MR renal angiography or Doppler ultrasonography is not recommended, except in cases with clinical suspicion of renal artery stenosis*.

### Blood pressure assessment

After hospital discharge, office BP or additional home BP measurements should be conducted at a 1- to 2-week follow-up visit to detect the early response in some patients, even though most patients require several weeks or months until BP reduction becomes apparent. HBPM and ABPM have proven benefits over office BP measurement to ensure the diagnoses of white-coat hypertension and masked hypertension and are better correlated with target organ damage [70]. Moreover, ABPM is more valuable than HBPM in detecting “morning surge” and “nocturnal hypertension”, which are strongly associated with cardiovascular events [71, 72]. Masked uncontrolled HT, including nocturnal HT, is more frequent in Asian and Thai populations [19], possibly due to higher salt intake and salt sensitivity. Reduced dipping and rising nocturnal BP profiles have been found in 74% of Thai hypertensive subjects compared to 47% in Japanese hypertensive populations [19]. Considering the usefulness of RDN in 24-h sustained BP controls [18], it is reasonable to consider using ABPM after RDN. Information obtained from ABPM after RDN will confirm the effectiveness of RDN on the improvement of the 24-h BP profile and could further assist in antihypertensive medication adjustment.

According to the second-generation RDN trials, at least one ABPM value should be obtained at 3–6 months following RDN to evaluate the 24-h BP response. If possible, the annual follow-up of ABPM should be obtained in addition to office BP and home BP measurements for assessing the long-term durability of RDN.

### Renal function study: serum creatinine and estimated glomerular filtration rate

There is concern that RDN might cause a decline in renal function. An immediate deterioration of renal function following RDN may be attributed to many factors, such as renal artery injury, distal renal artery embolization, or contrast-induced nephropathy. In many sham-controlled trials, there was no significant change in serum creatinine between the RDN and control groups after the procedure. A meta-analysis including 48 cohorts totaling 2381 patients showed no significant difference in eGFR for up to 9 months [73]. From the Global SYMPLICITY Registry, the observed eGFR decline within 3 years in the RDN group was within the range of expected decrease in patients with severe hypertension [22]. However, the increase in the amount of contrast media used to achieve complete denervation in current practice, as much as an average of 270 ml in the SPYRAL ON MED trial, might cause greater renal damage than previously reported [52]. Therefore, renal function assessment should be acquired on the next day and again 1–2 weeks after the procedure.

### Follow-up of renal artery imaging: CT or MR renal angiography or Doppler ultrasonography

There was a concern that RDN may injure the renal artery endothelial lining, inducing subsequent renal artery stenosis. However, vascular complications, including access site complications, renal artery dissection, or stenosis, are rare after RDN, even in long-term follow-up. By using optical coherence tomography and intravascular ultrasound to detect local tissue damage following RDN, the incidence of microinjury was found to be low without clinical impact in one study [74]. A meta-analysis of 14 studies showed that only one out of 511 individuals had new significant renal artery stenosis after a median of 11 months following RDN [75]. Registry data over 3 years showed that 0.3% of renal artery stenosis and other clinical event rates were within the expected range for hypertensive patients [76]. Therefore, routine renal artery imaging may not be necessary and should be obtained only in patients with procedure-related renal artery injury or clinical suspicion of renal artery stenosis, as indicated by an unexplained worsening of renal function or deteriorating hypertension.

### Experience of the RDN operator

- *RDN should be performed by a well-trained and experienced operator*.

An inexperienced operator should undergo formal training or perform RDN under a proctor. In SYMPLICITY HTN-3, more than 50% of the operators had performed only two or fewer procedures during the trial, raising a concern that the inexperience of the operators might play a role in the negative outcome of this trial [63]. This concern was supported by the results from the Global SYMPLICITY Registry, revealing that significant BP reduction was achieved by experienced operators, together with a higher total number of ablations. As sham-controlled RCT studies and registries have shown the solid efficacy and safety of RDN, this treatment modality should not be restricted to clinical studies or centers of excellence as long as it can be performed by experienced operators [18]. RDN operators should understand the indications, contraindications, and procedure details clearly, while inexperienced operators should undergo a formal training program or perform RDN under proctor/experienced-operator supervision during their first few cases to ensure the safety and completeness of the denervation.

## Conclusion

Currently, there is a need for a novel treatment strategy to assist in BP control in difficult cases of hypertension. There is increasing evidence confirming the effectiveness and safety of RDN, mainly in resistant hypertension. Owing to the restricted budgets and limited availability of RDN in Thailand, the procedure should be considered only for certain groups of hypertensive patients, including those with refractory hypertension; resistant hypertension, especially with established clinical ASCVD or progressive target organ damage; or hypertension with multidrug intolerance or nonadherence. The routine use of RDN for the control of hypertension should not be performed at the present time.
